# Ferritin Increase in Hemochromatosis Subjects After Discontinuing Their Regular Maintenance Treatment: A Longitudinal Analysis Performed During the COVID-19 Imposed Hospital Lockdown

**DOI:** 10.1097/HS9.0000000000000770

**Published:** 2022-08-23

**Authors:** Maria P. Coutinho, Maria José Teles, Graça Melo, Marta Lopes, Delfim Duarte, Tiago L. Duarte, Júlia Reis, Ana Cláudia Martins, José Carlos Oliveira, Graça Porto

**Affiliations:** 1 Clinical Hematology, CHUP-Centro Hospitalar Universitário do Porto, Portugal; 2 Basic & Clinical Research on Iron Biology, i3S- Instituto de Investigação e Inovação em Saúde, Universidade do Porto, Portugal; 3 Hematopoiesis & Microenvironments, i3S- Instituto de Investigação e Inovação em Saúde, Universidade do Porto, Portugal; 4 Department of Onco-Hematology, Instituto Português de Oncologia (IPO)-Porto, Portugal; 5 Unit of Biochemistry, Department of Biomedicine, Faculdade de Medicina da Universidade do Porto, Portugal; 6 Clinical Chemistry Laboratory, CHUP-Centro Hospitalar Universitário do Porto, Portugal; 7 Department of Molecular Immunology and Pathology, ICBAS, Instituto de Ciências Biomédicas Abel Salazar, Universidade do Porto, Portugal

Hemochromatosis (HC) is a genetic disorder in which an uncontrolled intestinal iron absorption, due to defective hepcidin signaling, may lead to a progressive tissue iron overload.^1^ By far, the most common form of HC is due to *p.Cys282Tyr* homozygosity in the *HFE* gene, this being particularly prevalent in Caucasians, and clearly the most studied and well-defined iron overload genetic disorder.^2^ If not treated, HC may lead to disabling and life-threatening complications such as arthritis, diabetes, heart failure, hepatic cirrhosis, and hepatocellular carcinoma, although these complications are effectively prevented with an early diagnosis and appropriate treatment.^3^

Phlebotomy (venesection therapy) is the standard treatment for HC, being accepted for more than 60 years as the most effective way to prevent or reduce the disease-related morbidity and mortality.^4^ According to recent therapeutic recommendations of the Hemochromatosis International Taskforce,^5^ all patients homozygous for the *p.Cys282Tyr HFE* variant and with an iron overload phenotype should be treated with an intensive phlebotomy regimen of 400–500 mL per week until reaching a serum ferritin level <50 ng/mL, provided that there is no anemia.^5^ This treatment schedule assumes that each 500 mL phlebotomy withdraws approximately 250 mg of iron, which is subsequently released, in a compensatory process, from overloaded tissues (especially the liver). Intensive phlebotomy is completed once the body iron excess is fully removed. Following this induction phase, HC patients start a lifelong maintenance phase intended to prevent tissue iron reaccumulation. Maintenance treatment consists of regular phlebotomies programmed for each patient at the necessary frequency to maintain the serum ferritin between 50 and 100 ng/mL.^5^ Although there has never been a prospective controlled clinical trial to define the ideal regimen or frequency of the maintenance phlebotomies, the most commonly used schedule is one phlebotomy every 3 or 4 months in males or in females, respectively.^6^ This schedule is based on the frequencies normally recommended for healthy regular blood donors. The rate of iron reaccumulation, if treatment is discontinued, was once estimated as an increase of serum ferritin of 100 ng/mL per year,^7^ thus supporting the recommendation of keeping a regular program to maintain a low serum ferritin. To the best of our knowledge, no further studies corroborated this estimation, as it is not easy nor expected to follow-up HC patients without treatment to confirm the data. The contingencies imposed by the coronavirus disease 2019 (COVID-19) pandemic, however, provided a unique opportunity to address this question and test what is the impact of stopping maintenance phlebotomy treatments in HC patients, as this was imposed by the hospital lockdown policy.

Due to the COVID-19 pandemic restrictions, the CHUP Hematology Day Hospital was locked down from March 2020 to May 2020 and the HC patients regularly followed at this center had to interrupt their regular maintenance treatments, programmed every 2–6 months, depending on their iron status. After a 2-month period, patients were rescheduled to resume their treatments. Some patients still fell within their regular time schedules, but many of them, either because of hospital restrictions or by their own decision (fear of the hospital environment), delayed their treatments by periods that in some cases exceeded 1 year. This provided us with the unique opportunity to analyze the impact of the suspension time lapse, calculated as the number of days between the pre-lockdown (Pre-L) visit and the first post-lockdown (Post-L) visit, on the hematological counts, including hemoglobin (Hgb), the red blood cell indices, the reticulocyte hemoglobin content (Ret-He), and on the iron-related parameters (transferrin saturation and serum ferritin), all determined by the hospital standard routine methods in both visits. We complemented the study with analyses of hepcidin, erythropoietin (EPO), soluble transferrin receptor (sTfR), and erythroferrone (ERFE) in serum samples collected in the Post-L visit. EPO and sTfR were determined by validated standard methods at the CHUP Clinical Chemistry Laboratory and the levels of hepcidin and ERFE were measured by enzyme-linked immunosorbent assay (ELISA) using the Intrinsic Hepcidin IDx ELISA Kit and the Intrinsic Erythroferrone IE ELISA Kit (Intrinsic Lifesciences, La Jolla, CA), respectively. For comparisons, we measured the same analytical parameters in a control group of 44 volunteer healthy blood donors (20 males and 24 females, aged 18–61 y), the majority (28/44) being regular donors with an average time lapse of 172 days from the last blood donation, not significantly different from the average Post-L time lapse in patients. Patients and blood donors gave their informed consent to participate in the study in compliance with the specific procedures approved by the CHUP Ethical Committee and their samples processed respecting the principles of confidentiality according to the Helsinki Declaration.

A total of 57 HC patients (33 males and 24 females, aged 23–79 y), all homozygotes for the p.C282Y variant in HFE, are included in this study. We excluded patients with current inflammatory conditions, alcohol abuse or serum ferritin values above 170 ng/mL in the Pre-L visit. Patients were invited to participate in the study at the time of their first visit after the hospital lockdown, and the laboratory data from both the Pre-L and Post-L visits were retrieved from their clinical records. Of note, the Pre-L laboratory values were obtained in samples collected immediately before the patients´ regular 400 mL maintenance phlebotomy, ascertained as time lapse “zero” on the graphs in Figures. The patients’ time lapses without treatment ranged from 69 to 336 days in males and from 92 to 380 days in females. Data were analyzed using the Statgraphics Statistical Graphics System.

Hematological and iron-related parameters of the HC patients are shown in Suppl. Table S1 summarizing the data at the time of their last phlebotomy before lockdown (Pre-L), when available, and data at the first visit after lockdown (Post-L). These are compared with the same parameters in controls. On average, all parameters in HC were within the upper/normal range both in males and females, and their values did not change significantly from the Pre-L to the Post-L visit. At the individual level, however, serum ferritin showed a statistically significant change with time, the difference between the Pre-L and Post-L levels increasing with increasing time lapse (Figure 1A—*P* < 0.0001; *R*^2^ = 50%; cc = 0.70). Interestingly, the calculated linear regression equation (indicated in Figure 1) estimated an increase of 101 ng/mL per year, in accordance with what was originally predicted by Adams et al.^7^ Post-L serum hepcidin was also dependent on the time lapse (Figure 1B—*P* < 0.0001; *R*^2^ = 36%; cc = 0.60), positively correlating with the Post-L serum ferritin (*P* < 0.0001; *R*^2^ = 27%; cc = 0.52). In spite of the etiopathogenic definition of HC as an hepcidin insufficiency, its observed correlation with iron reaccumulation could be expected based on the evidence from animal models showing that the response to chronic iron challenge in *Hfe* deficient mice is only partially blunted.^8^ An alternative hypothesis to explain the correlation between ferritin and hepcidin is that HC patients could be particularly sensitive to signs of iron deficiency, with concomitant hepcidin downregulation in response to the erythroid stimulation induced by phlebotomies, in spite of normal iron parameters. This hypothesis is consistent with the observation of significantly higher EPO levels in both male and female patients in comparison to controls (see Suppl. Table S1 and also Suppl. Figure S1) and a negative impact of the time lapse on EPO levels (Figure 1C—*P* = 0.0037; *R*^2^ = 23%; cc = –0.48). In terms of the other parameters analyzed, there was no impact of the time lapse on serum iron, transferrin or transferrin saturation, nor on Hgb levels (Suppl. Figure S1A–D).

**Figure 1.**
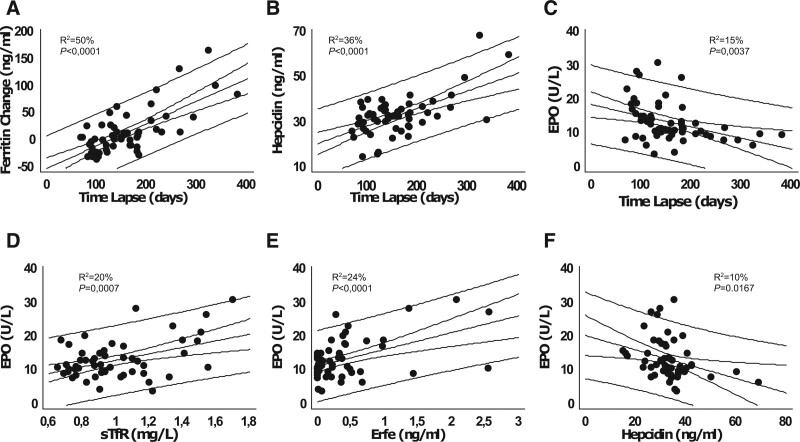
**The time lapse between phlebotomies impacts on iron metabolism and erythroid response of hemochromatosis patients in maintenance treatment.** The significant impact of the phlebotomy time lapse on serum Ft change (A), hepcidin (B), and EPO (C). The change in serum Ft in graph A was calculated as the difference between the Post-L and the Pre-L values. Pre-L samples had been collected immediately before a 400 mL maintenance phlebotomy, set as lapse time zero. Also shown the correlations between EPO and sTfR (D), ERFE (E), and hepcidin (F). Graphs display the correlation plots with the least square straight line, the 95% confidence bands, and the 95% prediction bands. The R squared values (*R*^2^) and significance levels (*P*) are indicated in each plot. The following regression equation predicts the increase in serum Ft with time lapse: (Post-L Ft)–(Pre-L Ft) = –50.3891 + 0.416522 × time lapse (d). EPO = erythropoietin; ERFE = erythroferrone; Ft = ferritin; Post-L = post-lockdown; Pre-L = pre-lockdown; sTfR = soluble transferrin receptor.

For better characterizing the EPO response of HC patients under phlebotomy treatment, we analyzed the relationships of EPO with other erythroid and iron-related parameters and found significant positive correlations with sTfR (Figure 1D: *P* = 0.0007; *R*^2^ = 20%; cc = 0.45) and ERFE (Figure 1E: *P* < 0.0001; *R*^2^ = 24%; cc = 0.49) and a negative correlation with hepcidin (Figure 1F: *P* < 0.0167; *R*^2^ = 10%; cc = –0.32). This “iron deficiency-like” profile contrasts with the consistently high serum transferrin saturation, high Hgb, and high Ret-He values (see Suppl. Table S1), supporting the notion of an inappropriate iron sensing in HC. The inappropriate EPO response of HC patients is also evident by calculating the difference between the observed EPO levels and the expected ones when corrected for the Hgb level, as described elsewhere.^9^ For that purpose, we estimated the expected values according to the regression equations of EPO and Hgb levels in the control group, as detailed in Suppl. Figure S2. The difference between the observed and expected EPO is also dependent on the time lapse (see Suppl. Figure S2). It is significantly higher with shorter time lapses, denoting an impact of the phlebotomy frequency on the EPO response, independently of systemic iron status.

Altogether these data support the notion that HC patients might have suppressed hepcidin not only because they cannot activate hepcidin expression but also because of persistent, EPO-induced ERFE production, more evident under the regular phlebotomy treatment, ultimately leading to an increased iron mobilization for erythropoiesis.

The results presented here raise some new questions, namely about the mechanisms modulating ferritin and hepcidin levels in the course of a regular phlebotomy program, and the role of EPO in that process. To the best of our knowledge, this is the first study supporting alterations in EPO regulation in HC patients. It could be expected that during regular phlebotomies, and once all hematological and iron parameters are within a normal range (see data on Suppl. Table S1), there would be no signals, neither iron deficiency nor hypoxia, to further stimulate EPO secretion. Nevertheless, we observed that in this cohort of HC patients in maintenance treatment, EPO was inappropriately high, in many cases at the levels observed in iron deficiency conditions. The mechanism underlying the putative higher sensitivity to iron deficiency in HC is still elusive. Future studies with animal models should clarify the complex crosstalk between HFE, EPO, ERFE, and iron mobilization. Of relevance to this question is the demonstration by Ramos et al^10^ that HFE is expressed in erythroid cells and that its absence exclusively from the hematopoietic compartment is sufficient to accelerate recovery from phlebotomy in Hfe deficient mice.

In conclusion, from a clinical point of view, the results obtained in this longitudinal study, performed in the context of the COVID-19 imposed hospital lockdown, support the importance of keeping a regular phlebotomy treatment in HC as a good strategy to maintain serum ferritin within the recommended desirable levels. We may speculate that the maintenance of regular phlebotomies in HC constitutes an effective and physiological means to downregulate hepcidin, while maintaining the iron mobilization to support an effective erythropoiesis and thus maintaining stable ferritin levels. From a more fundamental point of view, these results point toward new directions for better understanding the HC pathophysiology, namely the search for novel mechanisms underlying the putative higher sensitivity to iron deficiency in these patients.

## AUTHOR CONTRIBUTIONS

MPC, MJT, and GP conceived and designed the study, analyzed the results, and wrote the article. GM recruited patients, obtained informed consent, and collected and processed samples for analyses. ML, DD, TLD, JR, ACM, and JCO performed the laboratory analyses, discussed results, and reviewed the article.

## DISCLOSURES

The authors have no conflicts of interest to disclose.

## SOURCES OF FUNDING

This work was funded by National Funds through FCT - Fundação para a Ciência e a Tecnologia, I.P. - under the project UIDB/04293/2020. Hepcidin and erythroferrone ELISA kits were supplied by Intrinsic LifeSciences under the “First Annual Intrinsic LifeSciences Research Award, honoring Mark Westerman”.

## Supplementary Material


