# Supplementary material for: Ferritin Increase in Hemochromatosis Subjects After Discontinuing Their Regular Maintenance Treatment: A Longitudinal Analysis Performed During the COVID-19 Imposed Hospital Lockdown
Source: Hemasphere. 2022 Aug 23;6(9):e770. doi: 10.1097/HS9.0000000000000770 (PMC9400946; doi:10.1097/HS9.0000000000000770)
Supplement: Supplementary file 1 [file hs9-6-e770-s001.pdf]

Supplementary Table 1: Hematological and iron related parameters of the HC patients in maintenance treatment at the time of their last phlebotomy (Pre-lockdown) and first visit after lockdown (Post-lockdown) in comparison with the same parameters in a control group of regular blood donors

	**CONTROLS**		**HEMOCHROMATOSIS PATIENTS**					
	**Males**	**Females**		**Males**			**Females**	
	(n=20)	(n=24)		(n=33)			(n=24)	
**Age (years)***	40 (18-61)	39 (18-60)		53 (23-80)			59 (30-79)	
**Body Mass Index****				26±4			24±3	
			Pre-Lockdown	Post-Lockdown	*significance level* ***	Pre-Lockdown	Post-Lockdown	*significance level* ***
**Hemoglobin (g/dl)**	15.0±0.9	13.7±1.1	16.3±1.1	15.6±1.2	*P=0.0038*	14.1±1.3	13.6±1.0	*n.s.*
**Red Blood Cells ($x10^9$/L)**	5.1±0.3	4.5±0.3	5.1±0.4	5.0±0.4	*n.s.*	4.5±0.3	4.4±0.3	*P=0.0438*
**Mean Corpuscular Volume (fL)**	86.9±3.6	90.5±4.4	91.3±4.8	92.1±5.1	*P=0.0034*	91.7±4.0	93.0±3.5	*P=0.0318*
**Mean Corpuscular Hemoglobin (pg)**	29.6±1.6	30.2±1.6	32.3±1.8	32.1±1.8	*P<0.0001*	31.4±1.6	31.3±1.4	*P=0.0185*
**Ret-He (pg)**	33.5±1.6	34.0±1.8	34.8±1.4	34.6±1.8	*P=0.0229*	33.8±1.4	33.5±1.6	*n.s.*
**Iron (mg/dl)**	96±30	105±47	157±62	172±56	*P<0.0001*	136±38	130±48	*n.s.*
**Transferrin (mg/dl)**	270±60	303±57	218±26	209±30	*P<0.0001*	219±40	218±50	*P=0.0001*
**Transferrin Saturation (%)**	26±9	26±13	52±21	60±21	*P<0.0001*	45±14	45±19	*P=0.0002*
**Ferritin (ng/ml)**	139±103	59±80	98±43	114±64	*n.s.*	71±37	88±66	*P=0.0182*
**Hepcidin (ng/ml)**	28.9±30.0	14.7±15.1	n.a.	33.2±5.0	*n.s.*	n.a.	33.9±12.3	*P<0.0001*
**EPO (U/L)**	7.0±3.6	10.0±4.3	n.a.	13.8±6.6	*P=0.0001*	n.a.	13.2±4.9	*P=0.0199*
**Soluble Transferrin Receptor (mg/L)**	1.16±0.33	1.10±0.25	n.a.	1.01±0.24	*n.s.*	n.a.	0.98±0.29	*n.s.*
**ERFE (mg/ml)**	0.35±0.59	0.30±0.63	n.a.	0.55±0.71	*n.s.*	n.a.	0.18±0.24	*n.s.*

* average (range)
** average ± standard deviation
*** Comparisons with sex matched controls: The t-test was used for all parameters except for ferritin (in this case the Mann-Whitney W-test was used)
n.a. =not available; n.s.=not significant
