# Supplementary material for: Ferritin Increase in Hemochromatosis Subjects After Discontinuing Their Regular Maintenance Treatment: A Longitudinal Analysis Performed During the COVID-19 Imposed Hospital Lockdown
Source: Hemasphere. 2022 Aug 23;6(9):e770. doi: 10.1097/HS9.0000000000000770 (PMC9400946; doi:10.1097/HS9.0000000000000770)
Supplement: Supplementary file 2 [file hs9-6-e770-s002.pdf]

SUPPLEMENTARY FIGURE 1

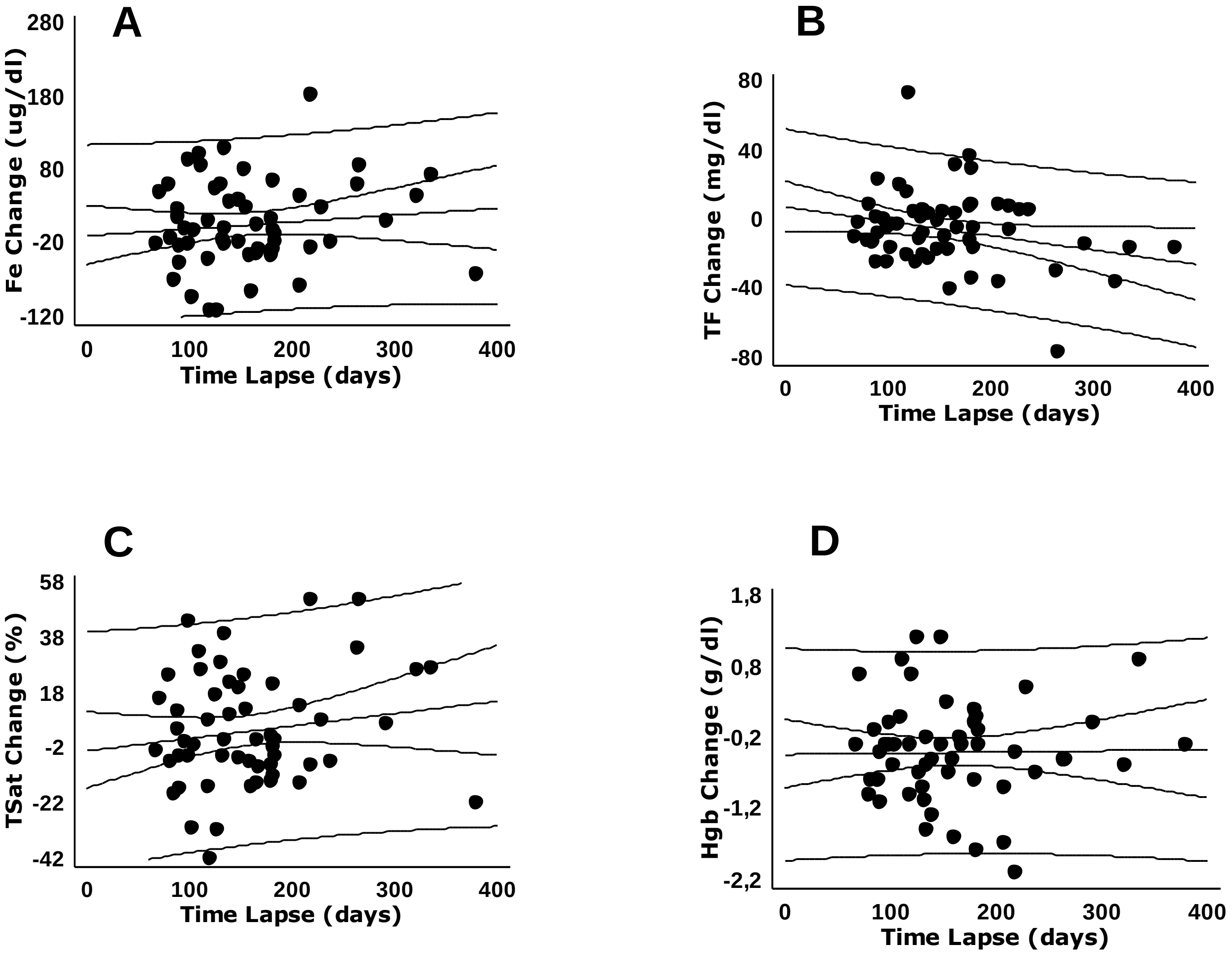

No significant impact of the phlebotomy time lapse on the change of serum iron (Fe) (A), transferrin (TF) (B), transferrin saturation (TSat) (C) and hemoglobin (Hgb) (D). The changes in parameters were calculated as the difference between the post-lockdown (Post-L) and the pre-lockdown (Pre-L) values. Pre-L samples had been collected immediately before a 400ml maintenance phlebotomy, set as lapse time zero. Graphs display the correlation plots with the least square straight line, the 95% confidence bands, and the 95% prediction bands.

SUPPLEMENTARY FIGURE 2

EPO expected values were calculated as a function of hemoglobin (Hgb), as previously published (*Beirão et 2004)*. For that purpose, we analyzed the correlation between EPO and Hgb in the group of healthy blood donors (R^2 =17%; P=0,005) and calculated the regression equations in males (blue open triangles in graph A) and females (red open triangles in graph A). The following equations were obtained: Y=19-0,8X in males and Y=27-1.3X in females. The plots are displayed in graph A including the least square straight lines (blue and red lines respectively for males and females). For comparison, graph A also displays the observed EPO values in hemochromatosis (HC) males (blue full circles) and females (red full circles) plotted against the respective hemoglobin values.

The differences between the observed and expected EPO levels were calculated and plotted against the lapse times (graph B) from last therapeutic phlebotomy (in HC patients; full black circles) or blood donation (in controls; open black circles) showing a significant correlation in HC patients (logarithmic best fitted model; R^2 24%; *P*=0,0005) but not in controls. The plot in graph B also contains the least square lines in controls (dashed black line) and in HC patients (continuous black line).

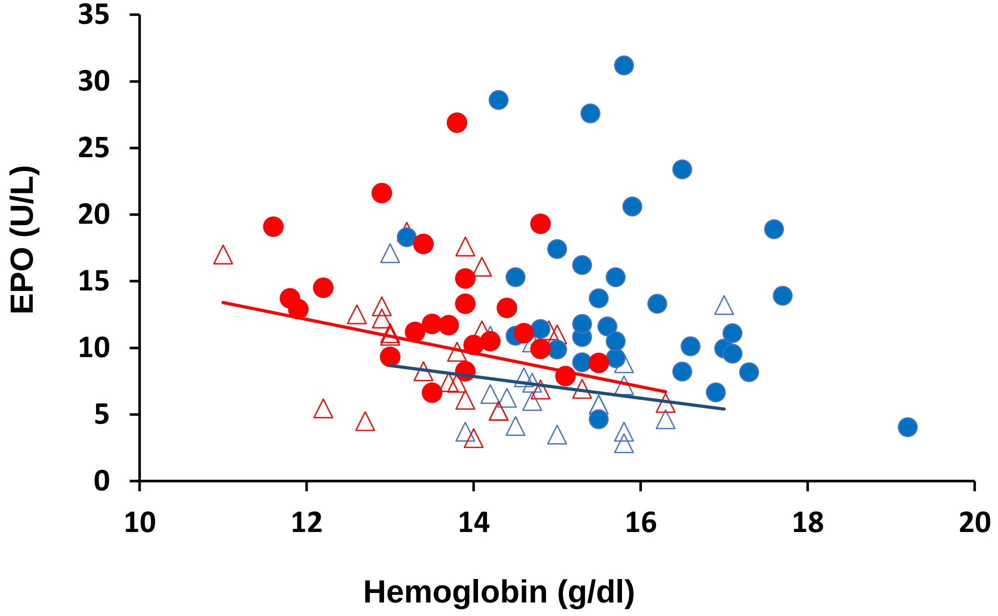
EPO (U/L)
35
30
25
20
15
10
5
0
10
12
14
16
18
20
Hemoglobin (g/dl)

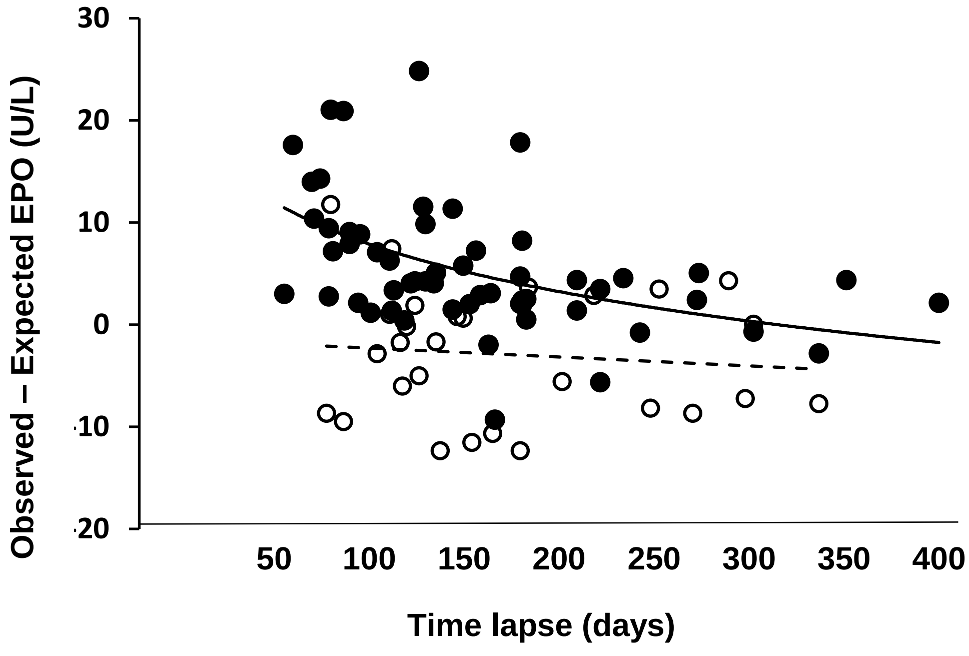
Observed – Expected EPO (U/L)
30
20
10
0
-10
-20
50
100
150
200
250
300
350
400
Time lapse (days)
